# Theoretical and Numerical Approaches for Determining the Reflection and Transmission Coefficients of OPEFB-PCL Composites at X-Band Frequencies

**DOI:** 10.1371/journal.pone.0140505

**Published:** 2015-10-16

**Authors:** Ahmad F. Ahmad, Zulkifly Abbas, Suzan J. Obaiys, Norazowa Ibrahim, Mansor Hashim, Haider Khaleel

**Affiliations:** 1 Institute for Mathematical Research, Universiti Putra Malaysia, Serdang, Selangor Darul Ehsan, Malaysia; 2 Department of Physics, Faculty of Science, Universiti Putra Malaysia, Serdang, Selangor Darul Ehsan, Malaysia; 3 Chemistry Department, Faculty of Science, Universiti Putra Malaysia, Serdang, Selangor Darul Ehsan, Malaysia; 4 Department of Engineering Science, Sonoma State University, Rohnert Park, California, United States of America; Northwestern Polytechnical University, CHINA

## Abstract

Bio-composites of oil palm empty fruit bunch (OPEFB) fibres and polycaprolactones (PCL) with a thickness of 1 mm were prepared and characterized. The composites produced from these materials are low in density, inexpensive, environmentally friendly, and possess good dielectric characteristics. The magnitudes of the reflection and transmission coefficients of OPEFB fibre-reinforced PCL composites with different percentages of filler were measured using a rectangular waveguide in conjunction with a microwave vector network analyzer (VNA) in the X-band frequency range. In contrast to the effective medium theory, which states that polymer-based composites with a high dielectric constant can be obtained by doping a filler with a high dielectric constant into a host material with a low dielectric constant, this paper demonstrates that the use of a low filler percentage (12.2%OPEFB) and a high matrix percentage (87.8%PCL) provides excellent results for the dielectric constant and loss factor, whereas 63.8% filler material with 36.2% host material results in lower values for both the dielectric constant and loss factor. The open-ended probe technique (OEC), connected with the Agilent vector network analyzer (VNA), is used to determine the dielectric properties of the materials under investigation. The comparative approach indicates that the mean relative error of FEM is smaller than that of NRW in terms of the corresponding S_21_ magnitude. The present calculation of the matrix/filler percentages endorses the exact amounts of substrate utilized in various physics applications.

## INTRODUCTION

Recently, plant fibres, such as those originating from species readily found in tropical areas (e.g., flax, OPEFB and jute), have attracted significant interest for use as a reinforcing element in bio-composite materials. Fibres from different plants have almost identical amounts of cellulose (44.4%) and hemicelluloses (30.9%) in addition to lignin and pectin (14.2%). These fibres remain an underused resource [1]. Lignin, which is the most important component of plant fibres, serves as an intercellular adhesive and has been shown to efficiently impact the frame, morphology and electrical conductivity of the fibre. The hardness of fibre-reinforced composite materials can be affected by several factors such as the intrinsic properties of the matrix, temperature, and the strength of the fibre ligament [2]. It has been shown that the thermal response of different fibres depends on their composition, while the cellulose structure enhances matrix adhesion and the mechanical properties of the fibre [3]. Several applications rely on natural fibres as reinforcements for thermoplastics and injection-moldable materials because of their high mechanical performance, low density and reduced impact on the environment [4–7]. Furthermore, these composites are recommended for use in electrical applications [2]. In this capacity, the dielectric constant, volume resistivity and loss factor are the important parameters of the polymer and fibre materials [8]. Dielectric studies of fibre-filled composites have linked the polarizability of the material with the amount of filler [2]. The electromagnetic interference shielding properties of plant rubber and ethylene-vinyl acetate mixed with carbon black and short carbon fibres have shown a dependence on fibre loading at fixed fibre content. A survey of the literature has revealed several studies concerning the electrical properties of filler-reinforced thermoset composites [9]. Natural fibers have further possibilities in waste management applications because of their biodegradability, as they can be utilized in highly functional composite samples in combination with biodegradable thermoplastic polymers [10]. The dielectric properties of pineapple reinforced polyethylene have been analyzed by Jayamol et al. [11]. These authors confirmed that increases in the dielectric constant of the composite directly correlated with increased fibre loading. Furthermore, inorganic polyester insulators have been utilized in electrical devices as dielectric substrates, insulators, embedding materials and common coatings [2]. Wollerdorfer et al. [12] have investigated the impact of fibre length distribution upon composite tensile strength for different types of polymer matrices. Other authors [8] have studied the mechanical properties of high-density polyethylene reinforced with continuous henequen fibres.

The implementation of materials in the microelectronics, microwave and radar industries requires accurate information regarding material properties such as the reflection (*R*) and transmission (*T*) coefficients. Microwave measurement techniques have been developed and proposed for the determination of the S-parameters [13]. In the T/R waveguide method, a sample with a fixed length is placed in a waveguide device, and the scattering parameter is determined using a vector network analyzer (VNA) at a frequency of 8-12GHz [14,15]. In this work, the network analyzer was calibrated using the standard full two-port calibration method (ECAL). Cross-section material samples are used in these measurements, which are the same as that of the transmission line, and a sample with a uniform cross-section is selected such that the dominant mode analysis is sufficiently precise for measuring the material constants. The relative permittivity of the samples is measured using the open-ended coaxial probe method based on algorithms for the characterization of solid materials. This experimental technique is based on the reflection coefficient measurement of the samples under test. FEM numerical techniques were used to simulate a double rectangular waveguide propagating a TE_10_ wave [16]. The radio wave (RF) technique was performed for the wave propagation issue, and computer software then calculates the TR coefficients based on the boundary conditions of the RF module. This paper presents efficient compositions for an OPEFB-PCL composite, thereby providing an opportunity to understand the amount of substrate to be used for various applications, such as in the telecommunications and electronics industries.

## METHODOLOGY

### 1. Finite element method

**Fig 1:**
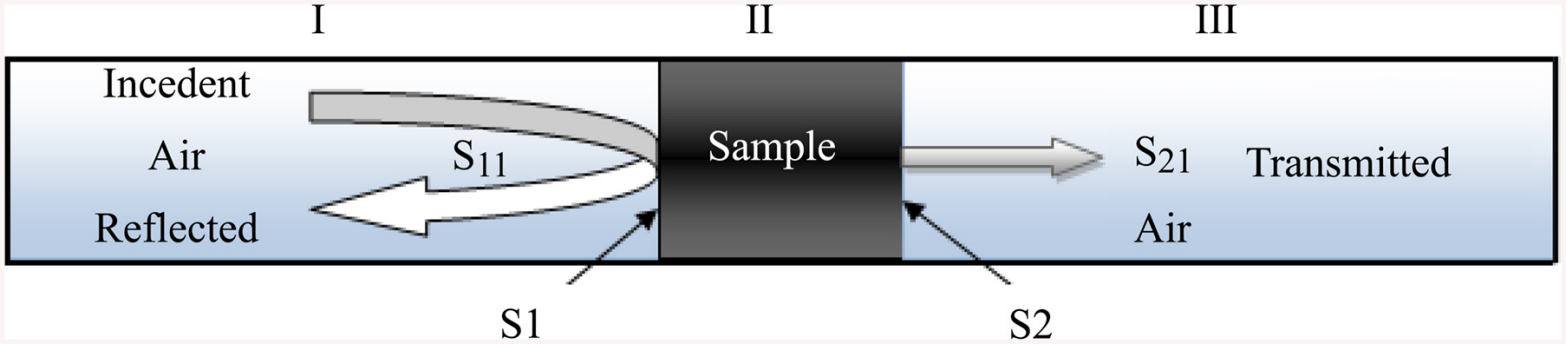
Incident, transmitted and reflected electromagnetic waves in a filled transmission line.

The FEM technique based COMSOL software is used to determine the exact transmission (S_21_) and reflection (S_11_) coefficients of the closed T/R rectangular waveguide. This model consists of a pair of rectangular waveguides with wave propagates in the transition between them. There are 16 boundaries; two of them for ports, another two represent the continuity on the sample surfaces while the rest are perfect electric conductors. For propagation problem, the harmonic propagation module of RF-electromagnetic waves is applied for the model. The problem is divided into three Regions: Region I (p<0), Region II (0≤p≤d) and Region III (p>d) for simpler analyses process. Fig 1 shows the waveguide excited by TE_10_ dominant mode and the transmission and reflection coefficients were measured at the reference plane (S_1;_ z = 0 and S_2_; z = d). By utilizing the waveguide vector modal functions, the transverse electromagnetic fields in both regions I and III are explained by [17].

The following equation has been solved for the electric field (E) vector inside the waveguide [17]

$$\nabla \;\times \;\left(\mu_{r}^{-1}\;\nabla \;\times \;E\right)\;-K_{0}^{2}\;\left(\varepsilon_{r}-\frac{j\delta }{\omega \varepsilon_{0}}\right)\;E\;=\;0$$ (1)

**Fig 2:**
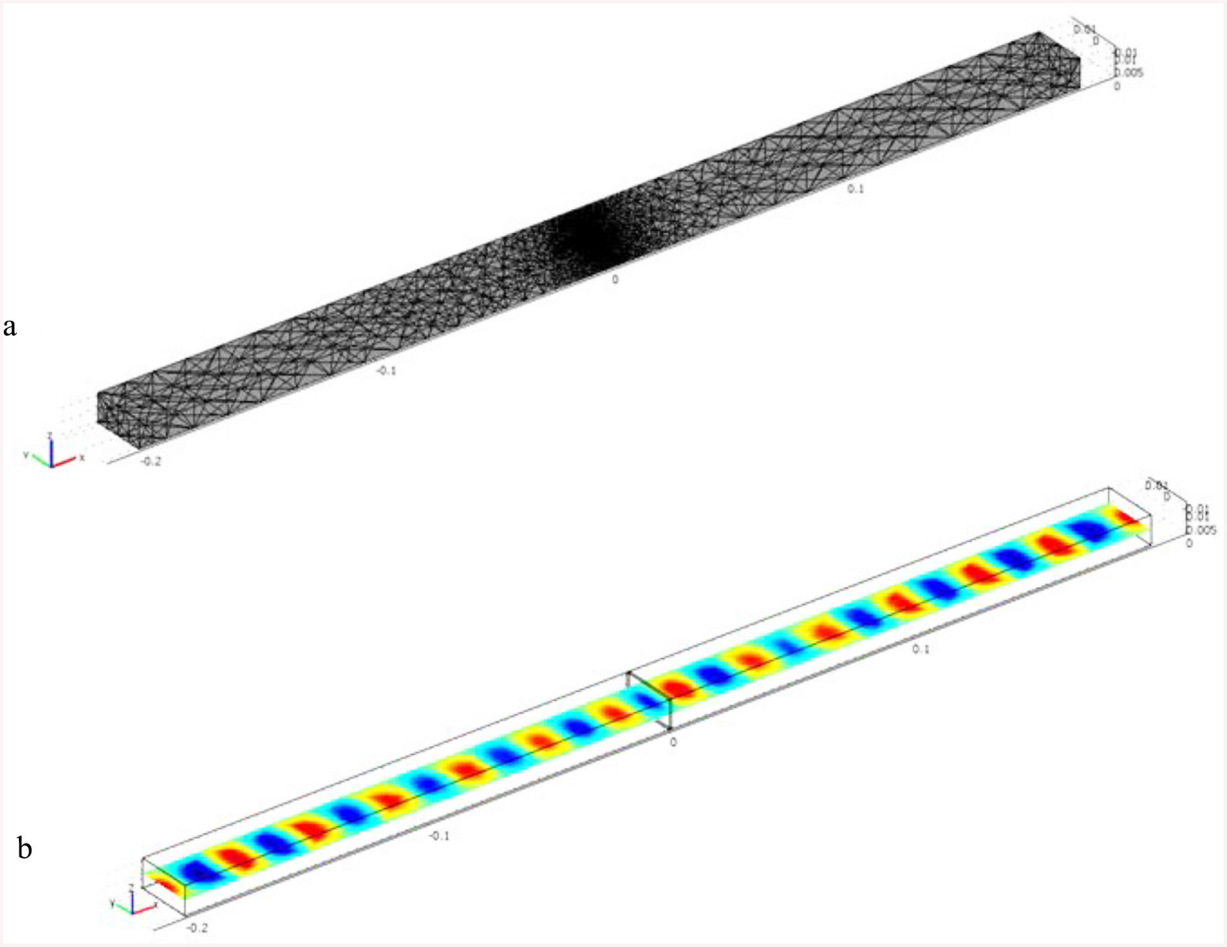
(a) Waveguide mesh carrying material sample (b) Electric field of a waveguide carrying sample.

Where *μ*_*r*_ is the complex permeability, *K*_*o*_ is the free space wave number, *j* is an imaginary unit, *δ* is the conductivity, *ω* is the angular frequency, *ε*_*r*_ is the relative permittivity, and *ε*_*o*_ is the permittivity of free space. The tetrahedron is used to describe the waveguide space because of its versatility in being able to conform for many other shapes. A fine mesh approximation type is accomplished due to its best accuracy for the waveguide carrying material sample. The mesh composed of triangles is generated from the cross-section of the waveguides, which is drawn in two dimensions with the aligned material sample. These triangles increase as the electrical density of the material sample increases. Subsequently, the 2D mesh is extruded into the depth dimension with a finite number of layers, producing triangular prism elements that divide into tetrahedrons, which generate the three-dimensional waveguide. Fig 2a shows the waveguide mesh with the material sample. The unknown field within each tetrahedron can be interpolated from each node value by a first-order polynomial. Fig 2b shows the normal electric distribution for an interpolated sample placed inside a waveguide of frequencies in the range of 8-12GHz. This figure shows that the electric field decreases as the excitation passes through the sample.

The electric field passing through Region II (E^II^) can be calculated from the FEM formulae, where the wave equation of E^II^ has the following form [18]

$$\nabla \times (\mu_{r}^{-1}\cdot \nabla \times {\vec{E}}^{II})-k_{0}^{2}\varepsilon_{r}{\vec{E}}^{II}=0$$ (2)

For effortless calculation, Eq (3) multiplied by a vector test function $\vec{T}(x,y,z)$ of the form $\vec{T}(x,y,z)=\overset{\wedge }{x}T_{x}+\overset{\wedge }{y}T_{y}+\overset{\wedge }{z}T_{z}$ Then, by integrating the outcome equation over the volume of Region II (V) along with elementary simplification, Eq (3) becomes

$$\underset{V}{∭}\left[\nabla \times \vec{T}\cdot \left(\mu_{r}^{-1}\nabla \times {\vec{E}}^{II}\right)-k_{0}^{2}\varepsilon_{r}{\vec{E}}^{II}\cdot \vec{T}\right]dv=-\iint_{S_{1}}\vec{T}\cdot \left(\overset{\wedge }{n}\mu_{r}^{-1}\nabla \times {\vec{E}}^{II}\right)ds$$ (3)

Where $\hat{n}$ is the normal unit vector of the lower boundary S_1_. Since $\nabla \times {\vec{E}}^{II}=-j\omega \mu_{0}{\vec{H}}^{II}$, Eq (3) becomes

$$\underset{V}{∭}\left[\nabla \times \vec{T}\cdot \left(\mu_{r}^{-1}\nabla \times {\vec{E}}^{II}\right)-k_{0}^{2}\varepsilon_{r}{\vec{E}}^{II}\cdot \vec{T}\right]dv=-\iint_{S_{1}}\vec{T}\cdot \left(\overset{\wedge }{n}\times (-j\mu_{r}^{-1}\omega \mu_{0})\cdot {\vec{H}}^{II}\right)ds$$ (4)

Where *μ*_0_ is the free space permeability.

Due to ${\left({{\vec{H}}^{I}|}_{S_{1}}\right)}_{\text{tan}}={\left({{\vec{H}}^{II}|}_{S_{1}}\right)}_{\text{tan}}\;\&\;{\left({{\vec{E}}^{I}|}_{S_{1}}\right)}_{\text{tan}}={\left({{\vec{E}}^{II}|}_{S_{1}}\right)}_{\text{tan}}$, then right integral in Eq (4) can be clarified as

$$\begin{array}{l} -\iint_{S_{1}}\vec{T}\cdot (\overset{\wedge }{n}\times (-j\mu_{r}^{-1}\omega \mu ){\vec{H}}^{II})ds=2(j\mu_{r}^{-1}\omega \mu_{0}).Y_{0}^{I}.\iint_{S_{1}}\vec{T}\cdot {\vec{e}}_{0}(x,y)ds\\ \;-(j\mu_{r}^{-1}\omega \mu ).\left(\sum_{P=0}^{\infty }Y_{P}^{I}\left(\iint_{S_{1}}\vec{T}\cdot {\vec{e}}_{P}(x,y)ds.\iint_{S_{1}}{{\vec{E}}^{II}|}_{S_{1}}\cdot {\vec{e}}_{P}(x,y)ds\right)\right) \end{array}$$ (5)

Substitute Eq (5) in Eq (4), yields

$$\begin{array}{l} \underset{V}{∭}\left[\nabla \times \vec{T}\cdot \left(\mu_{r}^{-1}\nabla \times {\vec{E}}^{II}\right)-k_{0}^{2}\varepsilon_{r}{\vec{E}}^{II}\cdot \vec{T}\right]dv=2(j\mu_{r}^{-1}\omega \mu ).Y_{0}^{I}.\iint_{S_{1}}\vec{T}\cdot {\vec{e}}_{{}_{0}}(x,y)ds\;\\ \;-(j\mu_{r}^{-1}\omega \mu ).\left(\sum_{P=0}^{\infty }Y_{P}^{I}\left(\iint_{S_{1}}\vec{T}\cdot {\vec{e}}_{{}_{P}}(x,y)ds.\iint_{S_{1}}{{\vec{E}}^{II}|}_{S_{1}}\cdot {\vec{e}}_{{}_{P}}(x,y)ds\right)\right) \end{array}$$ (6)

Where ${\vec{e}}_{p}\;,\;{\vec{Y}}_{p}$ are the p^th^ mode vector functions of the rectangular waveguide and modal admittance, respectively. For such complicated integral in Eq (6), an approximate solution for one volume of the tetrahedron V may facilitate the solution, where E in each tetrahedral element takes the form

$${\vec{E}}^{II}=\;\sum_{m=1}^{6}b_{m}\;\cdot {\vec{W}}_{m}$$ (7)

Where b_m_ represent the six complex amplitudes of E for the corresponding six tetrahedron edges and W_m_ is the corresponding m^th^ tetrahedron edge. Then substitute Eq (7) into Eq (6) and then integrating the result, gives

$$\begin{array}{l} \mu_{r}^{-1}\cdot \sum_{m=1}^{6}b_{m}\cdot \underset{V}{∭}\left[\nabla \times {\vec{W}}_{n}\cdot \nabla \times {\vec{W}}_{m}-k_{0}^{2}\varepsilon_{r}{\vec{W}}_{m}\cdot {\vec{W}}_{n}\right]dv=2(j\mu_{r}^{-1}\omega \mu ).Y_{0}^{I}.\iint_{S_{1}}{\vec{W}}_{n}\cdot {\vec{e}}_{{}_{0}}(x,y)ds\\ -(j\mu_{r}^{-1}\omega \mu )\cdot \sum_{m=1}^{6}b_{m}\cdot \sum_{P=0}^{\infty }Y_{P}^{I}\left(\iint_{S_{1}}{\vec{W}}_{n}\cdot {\vec{e}}_{{}_{P}}(x,y)ds.\iint_{S_{1}}{\vec{W}}_{m}\cdot {\vec{e}}_{{}_{P}}(x,y)ds\right) \end{array}$$ (8)

The above equation can be converted into a matrix form as follow [17]

$$\left[S_{el}]\cdot [N_{i}^{e}]=[v\right]$$ (9)

Where $N_{i}^{e}$, (*i* = 1, 2, 3…6) are the six complex amplitudes of the electric field associated with the six edges of the tetrahedron and the other elements are given by:

$$\begin{array}{l} S_{el}^{m,n}=\mu_{r}^{-1}\cdot \underset{V}{∭}\left[\nabla \times {\vec{W}}_{n}\cdot \nabla \times {\vec{W}}_{m}-k_{0}^{2}\varepsilon_{r}{\vec{W}}_{m}\cdot {\vec{W}}_{n}\right]dv+(j\omega \mu \mu_{r}^{-1})\cdot \sum_{P=0}^{\infty }Y_{P}^{I}\left(\iint_{S_{1}}{\vec{W}}_{n}\cdot {\vec{e}}_{{}_{P}}(x,y)ds.\iint_{S_{1}}{\vec{W}}_{m}\cdot {\vec{e}}_{{}_{P}}(x,y)ds\right)\\ v^{n}=2(j\omega \mu \mu_{r}^{-1}).Y_{0}^{I}.\iint_{S_{1}}{\vec{W}}_{n}\cdot {\vec{e}}_{{}_{0}}(x,y)ds \end{array}$$ (10)

These element matrices can be assembled over all the tetrahedron elements in the sample Region II in order to obtain a global matrix equation:

$$\left[S]\cdot [N^{e}]=[V\right]$$ (11)

The solution vector {*N* ^*e*^} of matrix Eq (11) is then used to determine the transmission and reflection coefficients at the reference plane, where [18]

$$T=\iint_{S_{2}}{\vec{E}|}_{S_{2}}\cdot {\vec{e}}_{{}_{0}}ds$$ (12)

$$R=\iint_{S_{1}}{\vec{E}|}_{S_{1}}\cdot {\vec{e}}_{{}_{0}}ds-1$$ (13)

Where E|_S1_ is the electric field of the surface area at the reference plane S_1_.

### 2. Nicholson-Ross-Weir (NRW) technique

To determine the electromagnetic properties of single-layered dielectric material based forward measured/simulated dielectric parameters, the well-known NRW algorithm is accomplished [19, 20]. This technique is comparatively easy to implement and it can accommodate materials with both dielectric and magnetic properties. MATLAB programming investigates the input/output data of the dielectric properties over the operating frequency range. The NRW method is proposed to calculate the S-parameters of a given sample. The transmission coefficient S_21_ at the measurement plane can be written as

$$S_{21}=\frac{T(1-\Gamma^{2})}{1-\Gamma^{2}T^{2}}$$ (14)

Where T is the transmission coefficient and *Γ* is the true reflection coefficient. The reflection coefficient can be derived using

$$S_{11}=\frac{\Gamma \left(1-{\text{T}}^{2}\right)}{1-\Gamma^{2}\Gamma^{2}}$$ (15)

When the S-parameters are extracted from the network analyzer, simultaneously solving the above equations provides the reflection coefficient as

$$\Gamma =\;X\pm \sqrt{X^{2}-1}$$ (16)

and the condition of |Γ| < 1 is imposed to determine the correct roots of this quadratic equation, and thus, the *X* parameter can be expressed as

$$X=\;\frac{S_{11}^{2}-S_{21}^{2}+1}{2S_{11}^{2}}$$ (17)

Therefore, the transmission coefficient can be written as

$$T=\frac{S_{11}+S_{21}-\Gamma }{1-(S_{11}+S_{21})\Gamma }$$ (18)

### 3. Sample Preparation

**Fig 3:**
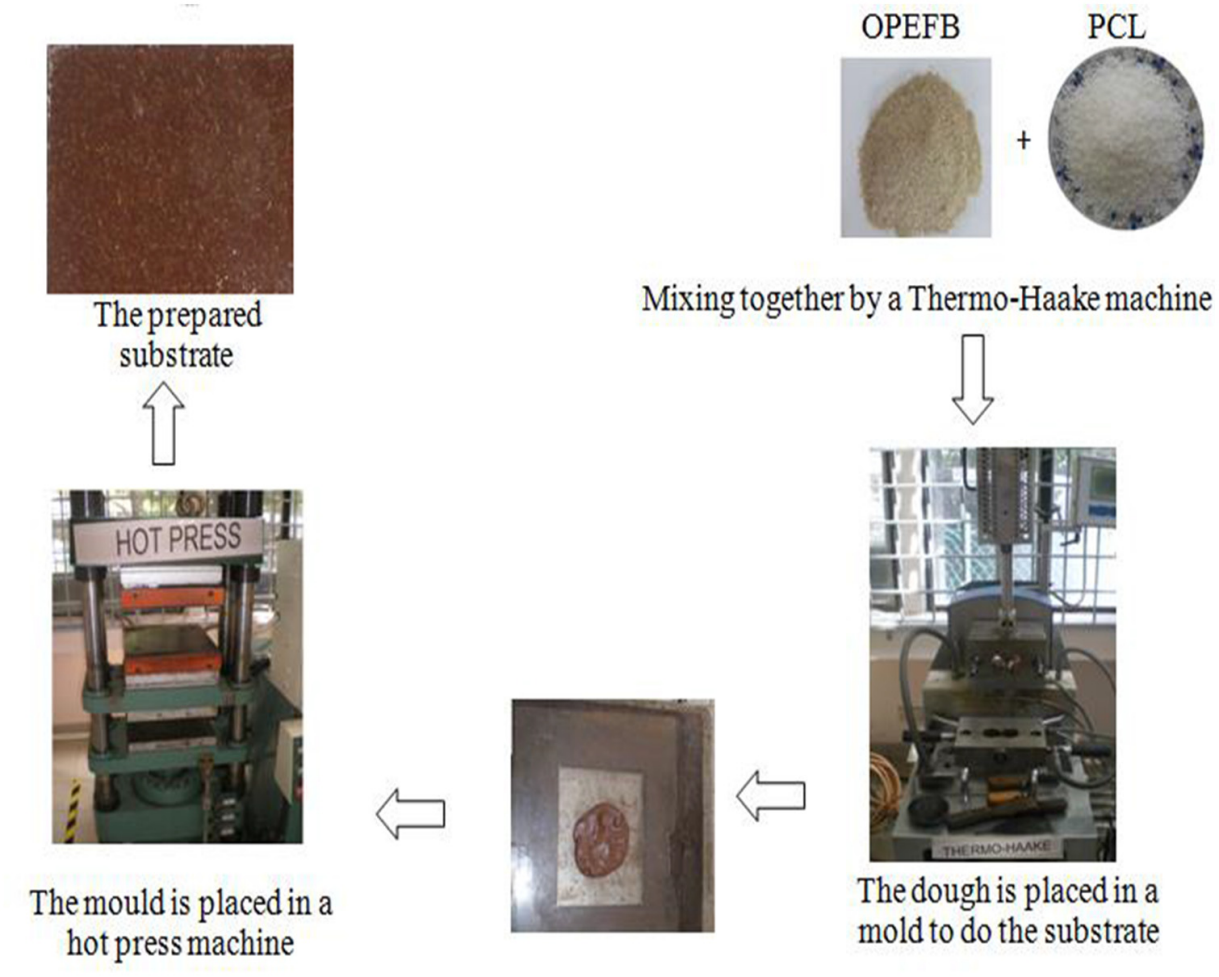
The Substrate preparation.

OPEFB fibre in this work was soaked in distilled water for 24 hours and then heated at about 80°C. This process was repeated twice. The filtered fibre was washed by acetone and then dried again in an oven to remove the wax layer of fibre. A grinded machine was used to grind fibre chains into small powder molecules and then sieved to sizes of 200μm. The compound of OPEFB-PCL was carried out in a Thermo Haake blending machine at 80°C with 50 rpm rotor speed for 20 minutes. The substrate of 1mm thickness was prepared by placing 10 g of the blend into a mold of 10×8cm^2^ dimensions. After that, OPEFB-PCL composites were preheated for 10 minutes with upper and lower plate. To reduce the void, a breathing time of one minute was allowed for bubble sand releasing. Finally, hot and cold pressed step of 110 kg/cm^2^ each was carried out for another 10 minutes each to obtain the required substrate. Fig 3 bellow illustrates the process of the substrate preparation.

## EXPERIMENTAL METHOD

**Fig 4:**
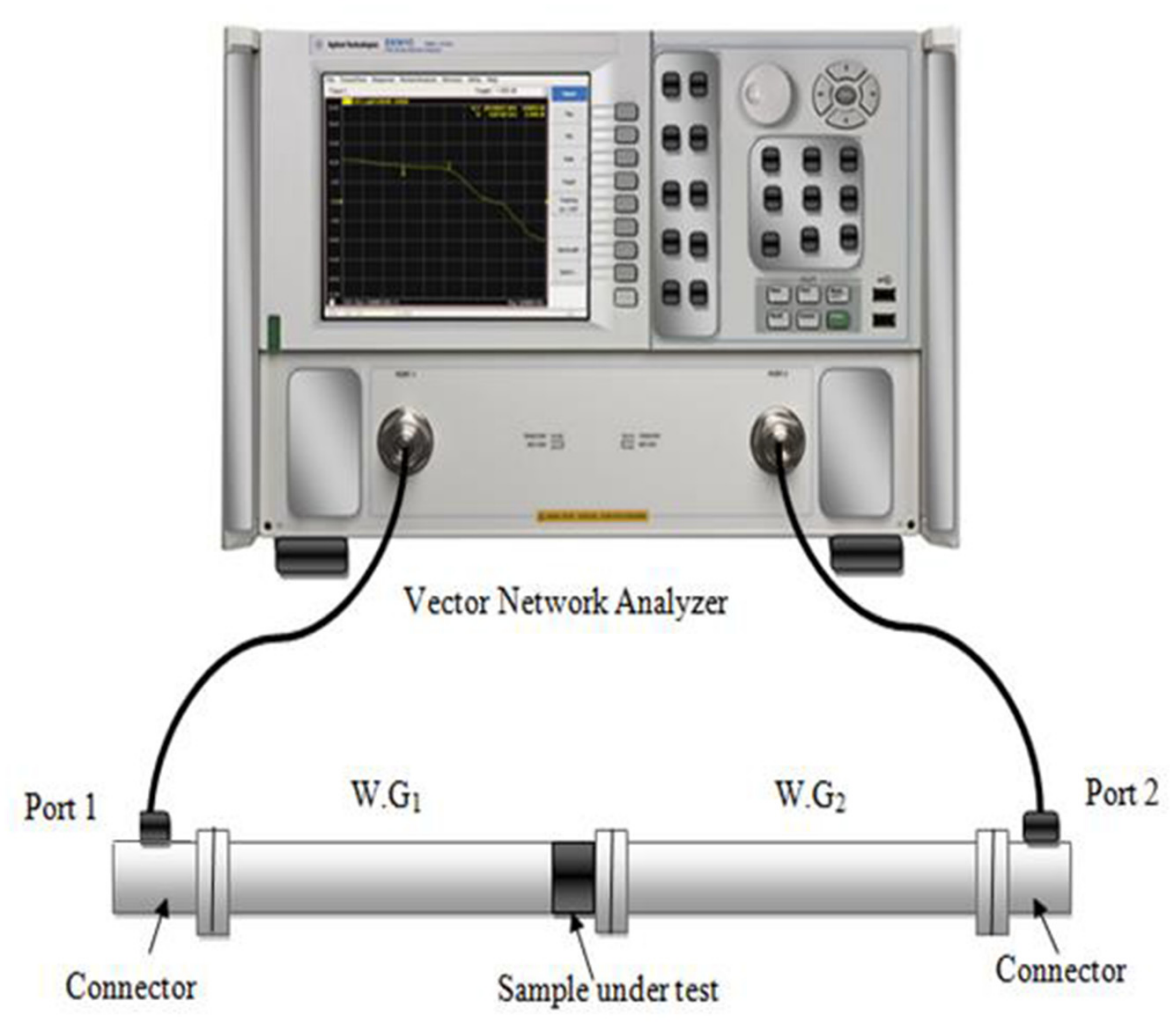
S-parameter measurement processes.

The network analyzer was calibrated using the standard full two-port Electronic calibration method (ECAL). The S-parameters measurements were carried out using closed T/R rectangular waveguide connected with two ports VNA as is clear in Fig 4. As recommended by the manufacturer, ECAL technique was applied to eliminate the systematic errors in S_21_and S_11_ measurements. The samples were cut to fit snugly into the rectangular waveguide. Fig 4 shows the measurements setup as well as the fitted sample inside the waveguide. Three different places of the obtained slab with same composition were measured to make sure the homogeneity of the prepared composites. The dielectric properties (ε′ and ε′′) of the samples were measured using an OEC connected with a VNA via a cable, where the probe was placed on the flat surface of the samples without an air gap. The measurement method was based on the input reflection coefficient of the coaxial line against the samples. The measurements of the ε′ and ε′′ of the samples were performed in room temperature at X-band frequency. The relative permittivity results obtained from the OEC are then used by the FEM technique for the simulation and subsequent calculation of the S-parameters of the samples used in this study. The S-parameter results obtained using the rectangular waveguide technique and the FEM were compared with those obtained using the NRW method.

## RESULTS AND DISCUSSION

### 1. Dielectric properties

As previously mentioned, the OEC technique is used to measure *ε*_r_, where (*ε*_r_ = *ε*'-j*ε*'') and*ε*' and *ε*'' are the real and imaginary parts of the permittivity, respectively. As shown in Table 1, the minimum dielectric constant of OPEFB-PCL composites is achieved using the highest percentage of fibre and the lowest percentage of polymer (63.8%OPEFB+36.2%PCL), whereas the maximum dielectric constant is achieved using the lowest percentage of fibre and the highest percentage of polymer (12.2%OPEFB+87.8%PCL). It is clearly observed that at all frequencies, the dielectric constant decreases as the fibre loading increases. Furthermore, the value of the dielectric constant, which depends on the polarizability of the molecules, decreases with increasing frequency for all of the composite samples. The polarizability of non-polar molecules arises from electronic polarization (in which an applied electric field causes a displacement of the electrons relative to the nucleus) and atomic polarization (in which an applied electric field causes a displacement of the atomic nuclei relative to one another). For polar molecules, a third factor is also involved: orientation polarization (in which an applied electric field causes an orientation of dipoles).Table 1 shows that the variation of the loss factor *ε*''with frequency is similar to that of the*ε*' values for all of the composite samples. The *ε*' and *ε*'' values for OPEFB are lower than those for the PCL. The reduction in the*ε*' and *ε*'' values is obtained by increasing the OPEFB content. The*ε*' and *ε*'' values of 12.2%OPEFB+87.8%PCLalmost overlap the other *ε*' and *ε*'' values due to the effect of pure OPEFB on the material absorption of OPEFB-PCL. In general, a higher *ε*'' is inversely proportional to the frequency. The results are obtained from the open-ended coaxial that was further used in the calculation of the S-parameters using the FEM method (S1 File).

**Table 1:** Relative permittivity values of various OPEFB-PCL compositions at 10 GHz.

Sample % wt OPEFB	Dielectric constant *ε*'	Loss factor *ε*''	Relative Permittivity *ε*_r_ = *ε*'-j*ε*''
PCL	2.844	0.367	2.844-j0.367
12.2%OPEFB+87.8%PCL	2.819	0.350	2.819-j0.350
23.8%OPEFB+76.2%PCL	2.682	0.333	2.682-j0.333
34.7OPEFB+65.3%PCL	2.550	0.314	2.550-j0.314
45%OPEFB+55%PCL	2.381	0.263	2.381-j0.263
54.7%OPEFB+45.3%PCL	2.243	0.202	2.243-j0.202
63.8%OPEFB+36.2%PCL	2.150	0.150	2.150-j0.150
OPEFB	1.866	0.061	1.866-j0.016

### 2. S-Parameters

**Fig 5:**
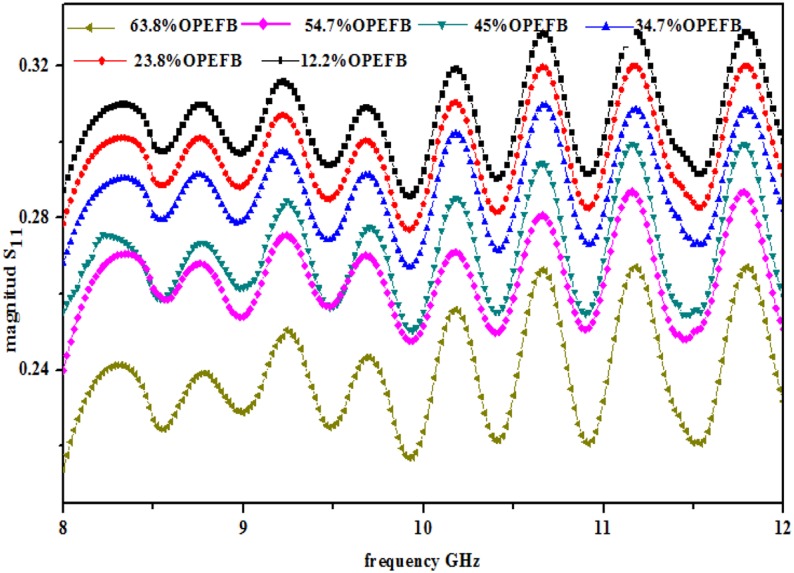
|S_11_| variation of samples inside rectangular waveguide at different filler percentage.

**Fig 6:**
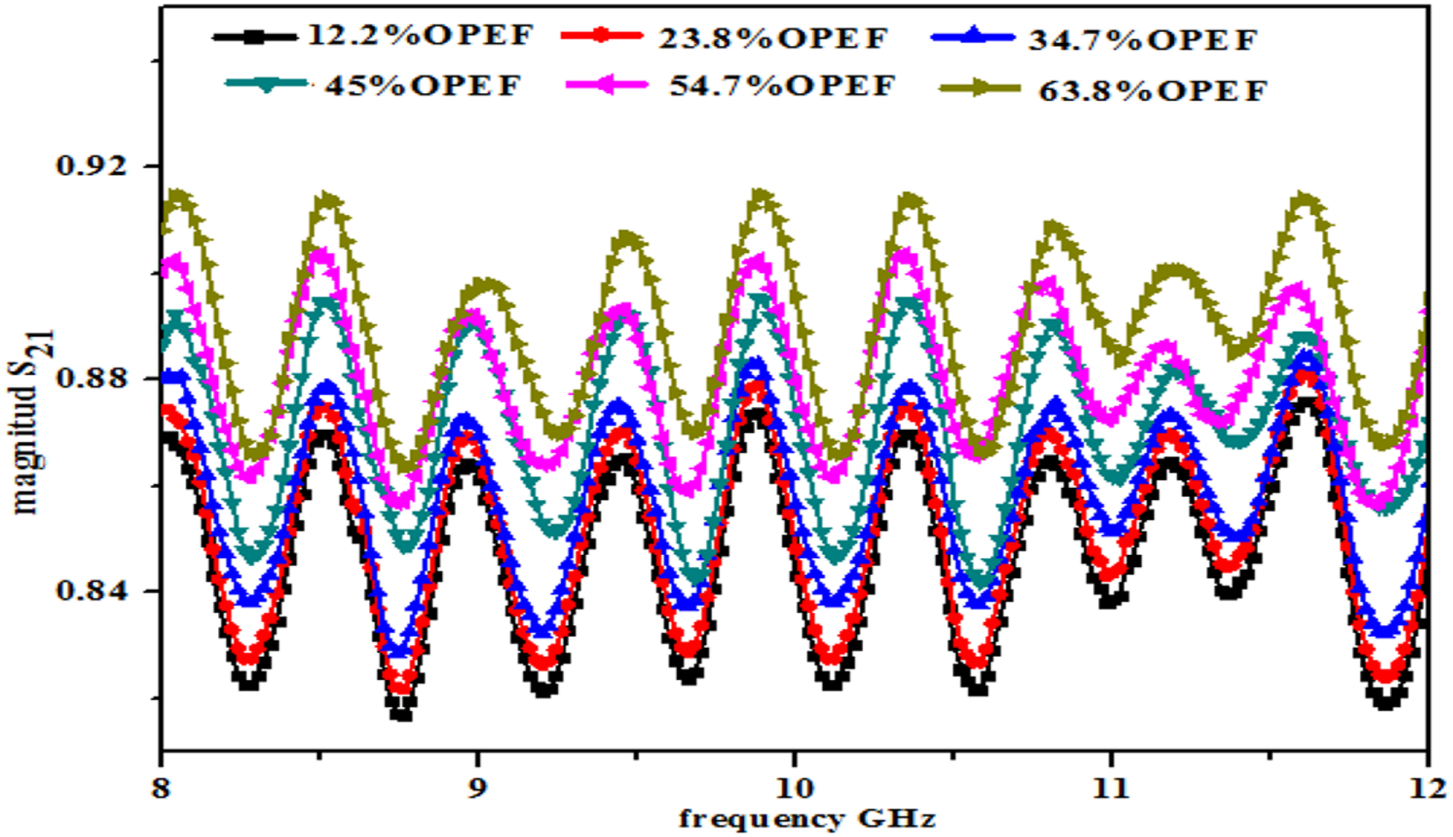
|S_21_| variation of samples inside rectangular waveguide at different filler percentage.

The variation in the magnitudes of the reflection coefficientS_11_ and transmission coefficient S_21_ for the OPEFB-PCL composites is shown in Figs 5 and 6, respectively. The sinusoidal-like wave form of the profile of S_11_was attributed to the impedance mismatch between the input impedance of the waveguide and the surface impedance of the sample and to the characteristic impedance of the coaxial cable [21], whereas the curve in the S_21_ measurements is due to the internal surface roughness of the waveguide. It is known that the air gap between the sample and the internal walls of the waveguide surface negatively affects the S_11_ and S_21_ results of the sample.

Fig 5 shows that an increase in the percentage of the OPEFB filler leads to a decrease in the S_11_ measurement results. This result indicates that a composition with a lower percentage of filler (12.2%) results in a higher S_11_ (0.288) value at 8GHz, whereas a higher OPEFB percentage (63.8%) provides a lower S_11_value (0.214) at the same frequency.

Fig 6 presents the rectangular waveguide results, which indicate that a higher percentage of filler (63.8%) provides a higher S_21_ result (0.908), whereas a lower percentage of OPEFB (12.2%) results in a lower S_21_ value (0.871). The presented results show that, in general, the S_21_ values are higher than the S_11_ values, which means that an increase in one value results in a reduction in the other value.

Table 2 shows the variation of |S_11_| and |S_21_| for all of the composite samples at various frequencies. As shown in this table, S_11_ increases and S_21_ decreases with increasing frequency for all of the composite samples.

**Table 2:** Variation of |S_11_| and |S_21_| for all OPEFB-PCL composite samples at different frequencies.

Samples OPEFB%	S_11_					S_21_				
	8 GHz	9 GHz	10 GHz	11 GHz	12 GHz	8 GHz	9 GHz	10 GHz	11 GHz	12 GHz
12.2	0.288	0.297	0.293	0.301	0.300	0.869	0.861	0.843	0.839	0.850
23.8	0.279	0.288	0.284	0.292	0.291	0.874	0.876	0.858	0.854	0.855
34.7	0.268	0.279	0.274	0.281	0.282	0.880	0.869	0.856	0.851	0.860
45.0	0.256	0.262	0.255	0.269	0.259	0.887	0.890	0.873	0.862	0.872
54.7	0.240	0.254	0.252	0.262	0.251	0.900	0.891	0.880	0.873	0.893
63.8	0.214	0.229	0.224	0.232	0.232	0.908	0.894	0.886	0.876	0.895

The restriction of (0 ≤ S_11_, S_21_ ≤ 1) must be considered for passive materials. The sinusoidal wave form in the profile of S_11_ is attributed to the impedance mismatch between the input impedance of the waveguide and the surface impedance of the sample and to the characteristic impedance of the coaxial cable [21]. The curve in the S_21_ measurements is due to the internal surface roughness of the waveguide.

### 3. Reflection Loss

**Fig 7:**
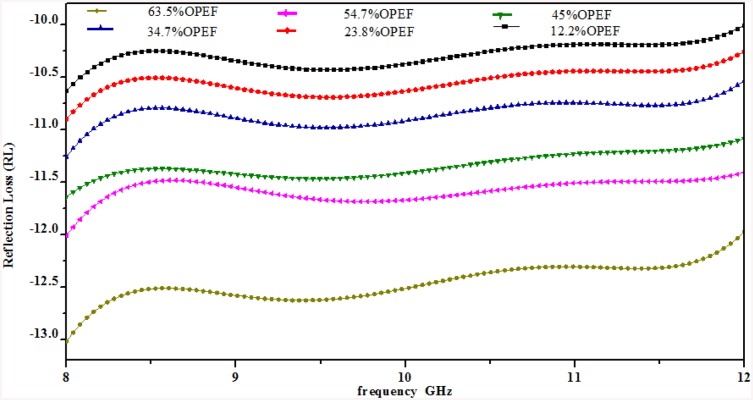
RL variation of OPEFB-PCL composites inside rectangular waveguide at different OPEFB filler percentage.

All of the samples with different weight percent loadings of OPEFB were prepared with a PCL matrix as reinforcement. The reflection loss (RL) was calculated from the S_11_ values obtained using a waveguide connected to a network analyzer using Eq (19), as shown in Fig 7.

$$RL=20\text{ log}|S_{11}|$$ (19)

The results presented in Fig 7 show a concomitant enhancement in the bandwidth and increased reflection loss with increasing OPEFB concentration. The variation of the RL values indicates the absorption effect or the minimum electromagnetic wave reflection (S2 File). The reduction of the intensity and frequency of the RL depends on the electronic properties, thickness of the samples and percentage of filler [22].

## COMPARISON OF THE RESULTS

**Fig 8:**
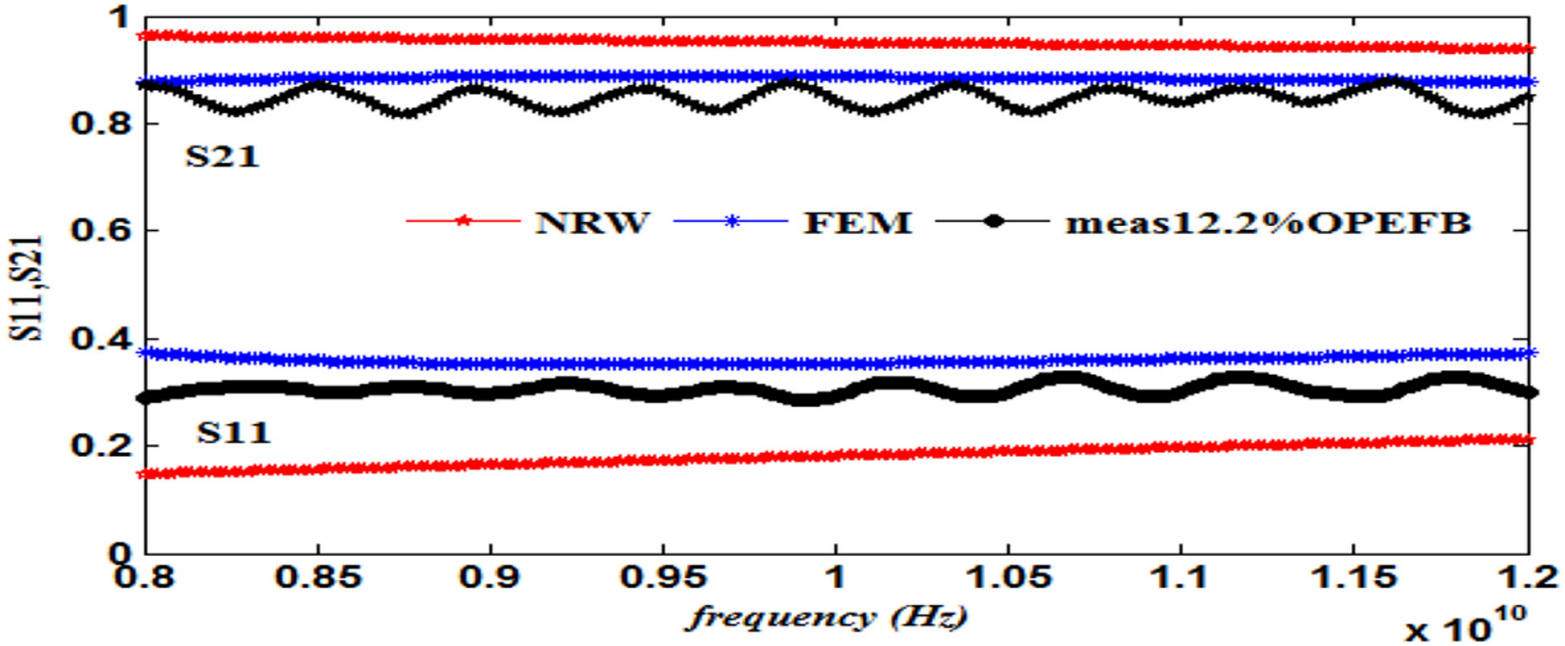
Measured, simulated and calculated S_11_ and S_21_ of (12.2%OPEFB +87.8%PCL).

**Fig 9:**
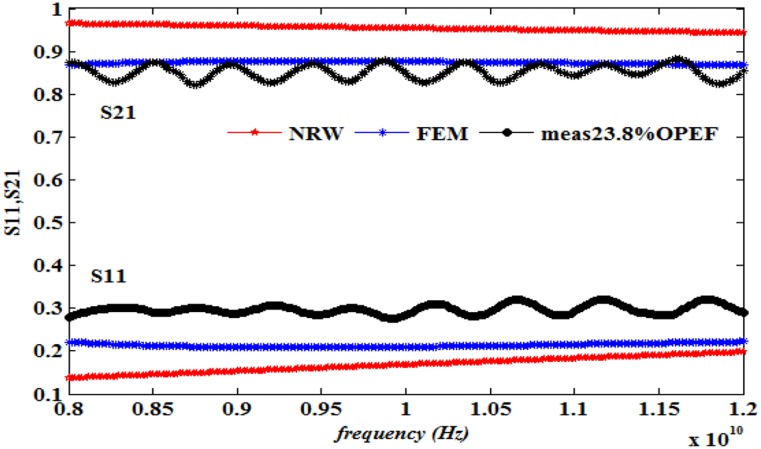
Measured, simulated and calculated S_11_ and S_21_ of (23.8%OPEFB+76.2%PCL).

**Fig 10:**
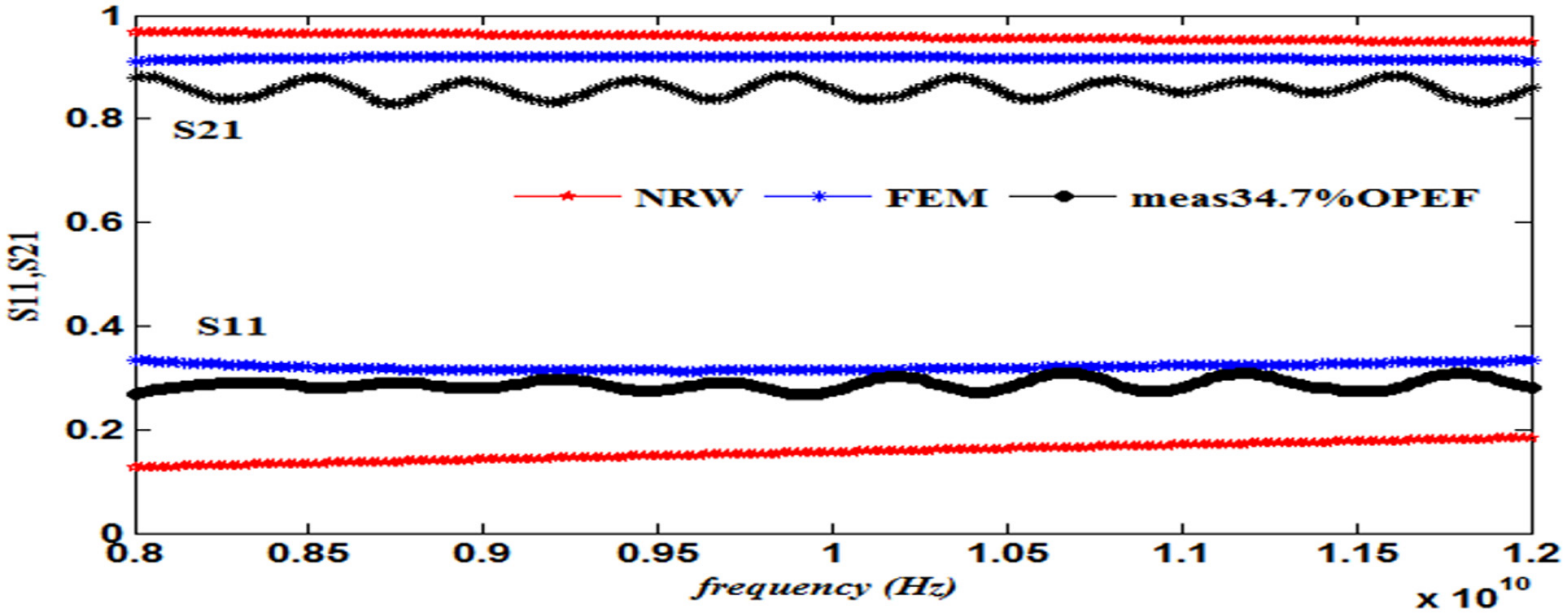
Measured, simulated and calculated S_11_ and S_21_ of (34.7%OPEFB+65.3%PCL).

**Fig 11:**
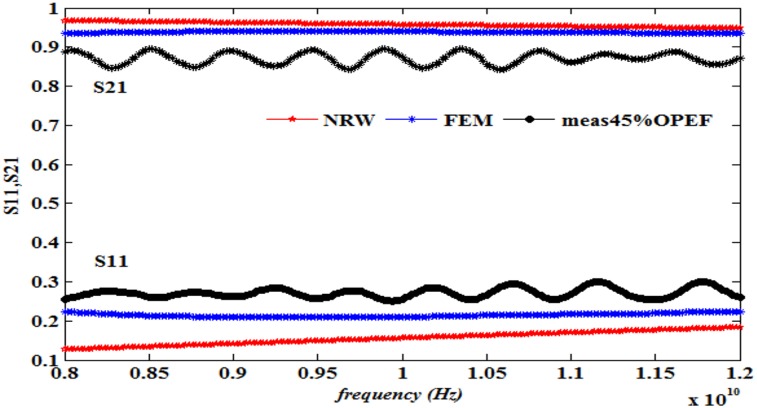
Measured, simulated and calculated S_11_ and S_21_ of (45%OPEFB+55%PCL).

**Fig 12:**
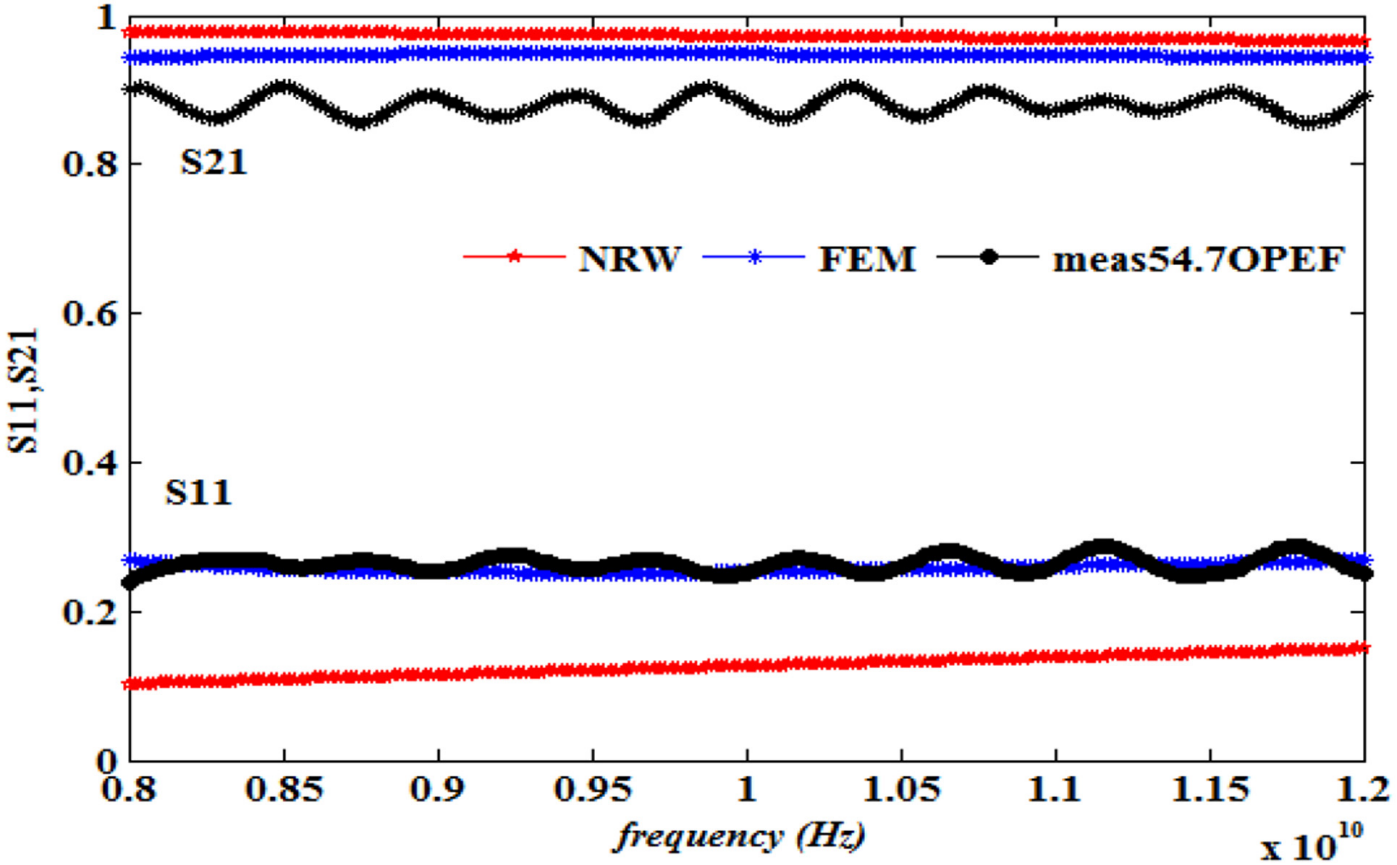
Measured, simulated and calculated S_11_ and S_21_ of (54.7%OPEFB+45.3%PCL).

**Fig 13:**
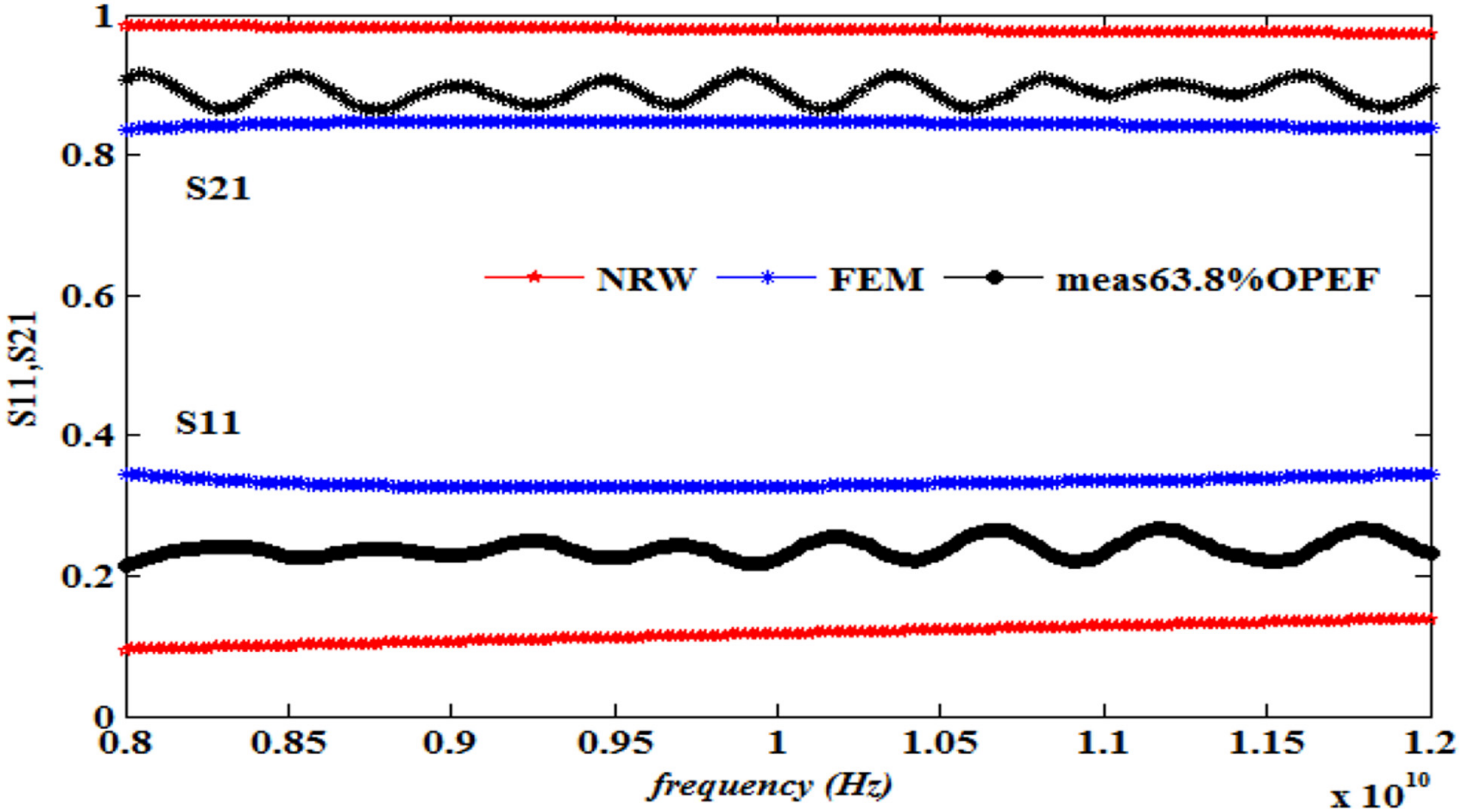
Measured, simulated and calculated S_11_ and S_21_ of (63.8%OPEFB+36.2%PCL).

This work also presents a comparison of the performance in terms of the calculated S_11_ and S_21_ using the NRW method (Eqs 14–18) and the FEM technique together with the measurements, as illustrated in Figs 8–13 (S3 File).

The dielectric properties obtained from the measurement of the composite samples were used as initial inputs to the NRW and FEM methods for the calculation of S_11_ and S_21_. The convergence analysis of the FEM method was conducted, whereas this analysis is not desirable for the NRW formulation. In general, the magnitude of S_11_ decreases with increasing filler percentage, which reflects larger S_21_ values. This relation gives the trend of the measured and simulated curves. The accuracy of the S_11_values can be determined by calculating the relative error with respect to the measurement data, as follows

$$\text{Re}lative\;error\;of\;S_{11}=\left|\frac{S_{11}(measurement)-S_{11}(NRW,\;FEM)}{S_{11}(measurement)}\right|$$ (20)

Note that the mean relative errors of S_21_ are calculated by replacing S_11_ with S_21_ in Eq (20).

The relative error values of both S_11_ and S_21_, along with the corresponding measurements presented in Table 3, show that of the two methods, the FEM provides values with greater accuracy than does the NRW method for the tested samples due to the input dielectric constant and loss factor. Although the NRW is based on a closed form, the numerical simulation showed better agreement because the calculations of the S-parameters using the NRW method involves several approximations that can be largely eliminated using the numerical simulation setup when samples of the fibre-reinforced polymers with different contents were tested.

**Table 3:** Relative errors of S_11_ and S_21_ in both the FEM and NRW methods for different samples.

Samples	Mean measured S-Parameters		Mean relative error in Eq (12)			
			S_11_		S_21_	
	S_11_	S_21_	NRW	FEM	NRW	FEM
**12.2%OPEFB+87.8%PCL**	0.3059	0.8827	0.1496	0.1359	0.0623	0.0249
**23.8%OPEFB+76.2%PCL**	0.2969	0.8800	0.1832	0.1411	0.0924	0.0790
**34.7OPEFB+65.3%PCL**	0.2871	0.8709	0.1723	0.2418	0.0165	0.0772
**45%OPEFB+55%PCL**	0.2710	0.8576	0.2037	0.2390	0.0664	0.0396
**54.7%OPEFB+45.3%PCL**	0.2631	0.8613	0.1631	0.1087	0.0467	0.0291
**63.8%OPEFB+36.2%PCL**	0.2381	0.8466	0.2129	0.1765	0.0610	0.0297
**Mean Relative Error**			0.1808	0.1738	0.0576	0.0466

Close inspection of Figs 8–12 reveals that both S_11_ and S_21_ obtained from FEM do not show increasing or decreasing pattern with increasing OPEFB concentration. In Figs 8–11, S_21_ values obtained from NRW and FEM are higher than measurements while in Fig 12, S_21_ value obtained from FEM is lower than measurements. Similar inconsistencies for S_11_ are also observed when comparing calculated values and measurements. These inconsistencies can be explained by looking at FEM Eqs (3)–(13) which show that both S_11_ and S_21_ are influences by *ε*' and *ε*'' values. In NRW formulations, the parameter *T* is also a function of *ε*' and *ε*'' values which is not shown in Eq 18 but can be found in [23]. As shown in Table 1, *ε*' and *ε*'' values change with increasing OPEFB concentration which lead to inconsistent trend of calculated S_11_ and S_21_.

## CONCLUSION

The results of this work are in contrast to the effective medium theory, which states that polymer-based composites with higher dielectric constants can be obtained using a lower filler value in combination with a higher dielectric constant host material. The dielectric constant values of the fibre-reinforced polymer system were found to be lower than that of the neat polymer due to the polarization exerted by the incorporation of fibres. Furthermore, the permittivity of the composites decreased as the filler values increased, and the lower the value of S_11_, the higher is the value of S_21_. Moreover, after introducing the fillers to the PCL matrix, S_21_increased as the filler content increased. The S-magnitude results of the composite for different filler percentages were successfully acquired using the waveguide technique, FEM and NRW methods. The open-ended coaxial method connected with a VNA in the 8–12 GHz frequency range was used to determine the relative permittivity of the materials under investigation. The FEM technique was found to generally be more accurate for determining the magnitudes of the reflection and transmission coefficients, S_11_and S_21_, of the composites placed in a closed T/R rectangular waveguide.

## Supporting Information

S1 File The related dielectric constant and loss factor file. (XLSX)

S2 File The reflection loss (RL) result file. (XLSX)

S3 File The simulated S_11_ and S_21_ result file. (XLSX)
